# Classification and characterization of hemocytes from two Asian horseshoe crab species *Tachypleus tridentatus* and *Carcinoscorpius rotundicauda*

**DOI:** 10.1038/s41598-019-43630-8

**Published:** 2019-05-08

**Authors:** Fangli Wu, Zhe Xie, Mingyan Yan, Qiongzhen Li, Jie Song, Menghong Hu, Youji Wang

**Affiliations:** 1 National Demonstration Center for Experimental Fisheries Science Education, Shanghai Ocean University, Shanghai, China; 2 International Research Center for Marine Biosciences at Shanghai Ocean University, Ministry of Science and Technology, Shanghai, China; 3 Key Laboratory of Exploration and Utilization of Aquatic Genetic Resources, Ministry of Education, Shanghai Ocean University, Shanghai, China; 4 Guangxi Academy of Fishery Sciences, Nanning, China; 5 Tianjin Era Biology Technology Co., Ltd, Tianjin, China

**Keywords:** Immunology, Molecular biology, Physics

## Abstract

In present study, transmission electron microscopy and flow cytometry were utilized to investigate the classification, characterization and immune functions of hemocytes from horseshoe crab, *Tachypleus tridentatus* and *Carcinoscorpius rotundicauda*. Three types of hemocytes were distinguished respectively: the granular cell, the semi-granular cell and the hyaline cell by transmission electron microscopy, while three hemocyte subpopulations (Gate 1 cell, Gate 2 cell, Gate 3 cell) were classified by flow cytometry. Hyaline cell was the major cell type with the highest nuclear-cytoplasmic ratio and granular cell and semi-granular cell showed lower ratios. Immune parameters of hemocytes in horseshoe crabs were investigated by flow cytometry. Different hemocyte subpopulations respond for diverse functions. Lysosomal contents and hemocyte mortality in Gate 3 cell subpopulation were higher than that in other subpopulations, while reactive oxygen species, phagocytosis and non-specific esterase, in Gate 1 cell subpopulation, were higher than those in other subpopulations. The hemocyte types between the two species had no significant differences in staining or morphology.

## Introduction

The horseshoe crab is a living fossil animal, belonging to Arthropoda, Chelicerata, Merostomata, Xiphosura. There are merely four species surviving in the world^1^ and three of them once have been discovered along the coasts of the East and South China Seas^2^, including *Tachypleus tridentatus* and *Carcinoscorpius rotundicauda*. *T*. *tridentatus* is vulgarly named as the Asian^1,3^, Chinese^4^ or Japanese horseshoe crab^5^ and *C*. *rotundicauda* is also called Asian^1,3^ or mangrove horseshoe crab^6^. The two species are distributed from East Asia and Southeast Asia^7–12^. In China, these two species live in the South China Sea and the East China Sea^13^, especially in some areas like Hong-Kong^14^ and the Beibu Gulf^15^.

In Japan, *T*. *tridentatus* has been regarded as a protected species owing to being endangered since 1928, but its population remains to be under threat^16^. The similar status also exists in China, for example, in Hong Kong the horseshoe crab population is also decreasing^15^. As an invertebrate animal, horseshoe crab only possesses of innate immune system^17–19^ and does not have adaptive immune system^20^. Hemocyte plays an important role in innate immunity as shown in many invertebrate animals^21–23^. Many studies on the blood of horseshoe crabs indicated that some elements in the blood are capable of eliminating some virus or foreign particles^24–26^, equivalently emphasizing the importance of hemocytes to immunity in horseshoe crab. Therefore, basic information on hemocytes in Chinese horseshoe crab *T*. *tridentatus* and mangrove horseshoe crab *C*. *rotundicauda* can provide insights for their health and immunology.

Three types of hemocytes have been identified in the two edible crabs *Cancer borealis* and *Cancer pagurus*^27^, the pearl oyster *Pinctada fucata*^28^, the Atlantic jackknife clam *Ensis directus*^29^, the Pacific oyster *Crassostrea gigas*^23^ and the zebra mussel *Dreissena polymorpha*^22^ using electron microscopy or flow cytometry. Using flow cytometric technique, the hemocyte immune parameters, such as total hemocyte counting (THC), phagocytosis (Pha), non-specific esterase (Est), lysosomal content (Lyso), hemocyte mortality (Hm) and reactive oxygen species (ROS) can be evaluated as well. Hemocyte parameters have been investigated in the pearl oyster *Pinctada fucata*^28^, the pacific oyster *Crassostrea gigas*^23^ and the zebra mussel *Dreissena polymorpha*^22^ by flow cytometry. The hemocyte types of cultivated Chinese horseshoe crab were classified into granule flattened cells, degranulated flattened cells and contract flattened cells by just light microscopy^30^. Also, the information on hemocytes of horseshoe crabs (*T. tridentatus* and *C. rotundicauda*) were investigated using optical microscopy and scanning electron microscopy, and the preliminary hemocyte classification has been proposed^31^. However, the inside features and immune parameters (THC, Pha, Est, Lyso, ROS, Hm) of hemocytes in the two Asian horseshoe crab species have not been reported well.

Some comparisons of hemocytes among/within species have been reported previously. The immune functions of hemocytes in different breeding generations of the swimming crab *Portunus trituberculatus* showed differences^32^. A comparative study of the hemocyte properties showed different functions between *Mytilus edulis* and *Aulacomya ater*^33^. A comparison between the mussel *Mytilus galloprovincialis* and the oyster *Crassostrea gigas* was made to ascertain which hemocyte possesses a higher resistance to the macrooganism infection^34^.

In the present study, transmission electron microscopy (TEM) and flow cytometry were used to view the interior morphology and classify the sub-populations of the hemocytes. In addition, THC, Pha, Est, Lyso, ROS and Hm were measured in two horseshoe crabs by flow cytometry. The study is the first comparison between Chinese horseshoe crab *T*. *tridentatus* and mangrove horseshoe crab *C*. *rotundicauda* on the immune parameters by flow cytometry. These new findings on horseshoe crab hemocytes can provide useful references for studying immunology of these two horseshoe crabs species.

## Results

### Transmission electron microscopy (TEM)

**Figure 1:**
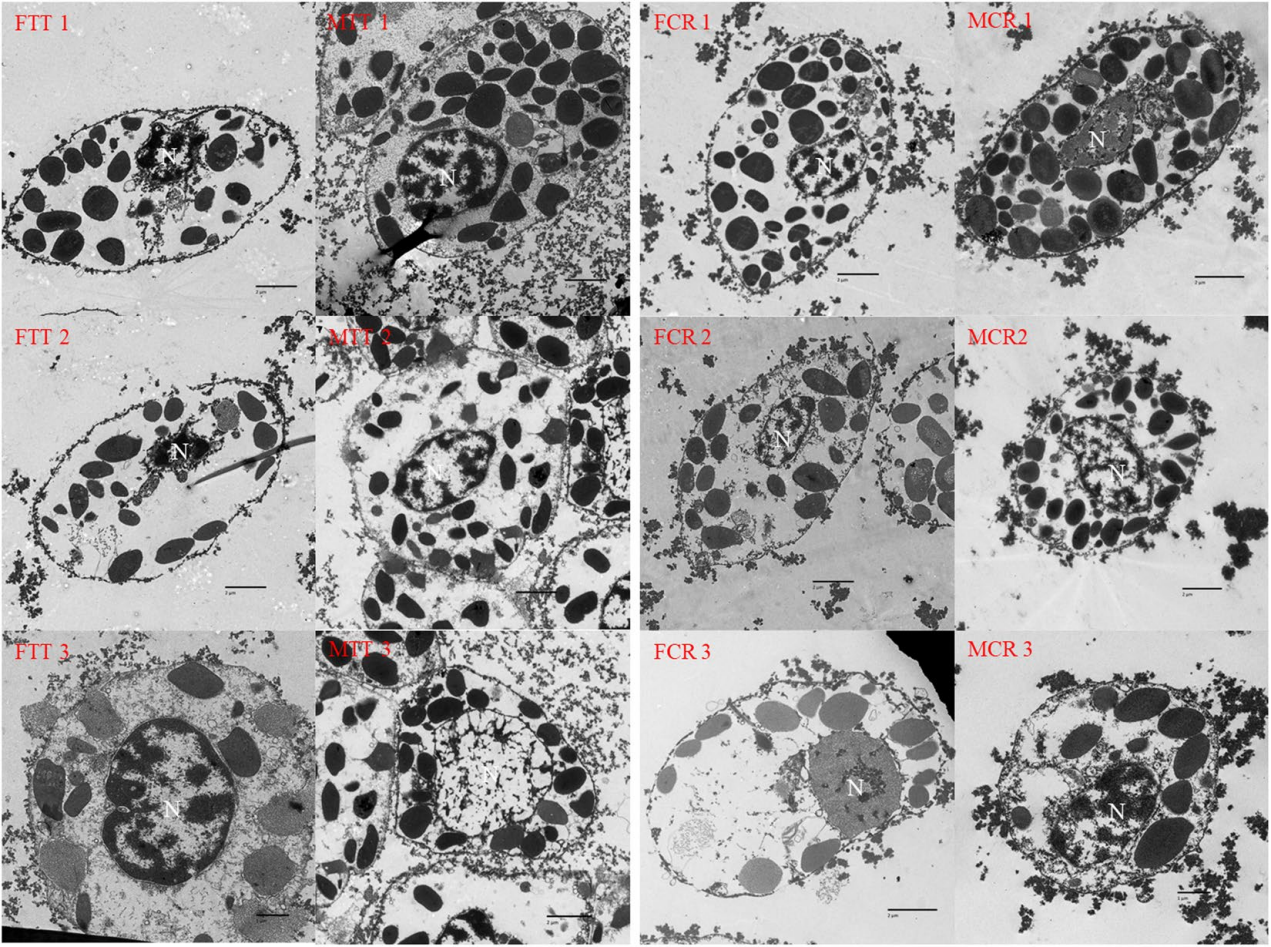
The morphology and ultrastructure of hemocytes of *Tachpleus tridentatus* and *Carcinoscorpius rotundicauda*. 1 indicate the granular cell; 2 indicate the semi-granular cell; 3 indicate the hyaline cell; FTT: the female *Tachpleus tridentatus* (FTT 1 × 2500, FTT 2 × 2500, FTT 3 × 4000); MTT: the male *Tachpleus tridentatus* (MTT 1 × 2500, MTT 2 × 2500, MTT 3 × 2500); FCR: the female *Carcinoscorpius rotundicauda* (FCR 1 × 2500, FCR 2 × 2500, FCR 3 × 3000); MCR: the male *Carcinoscorpius rotundicauda* (MCR 1 × 3000, MCR 2 × 2500, MCR 3 × 4000); N: nucleus.

The ultra-structures of the hemocyte were observed using TEM, dividing the cells into three types (granular cells: GCs, semi-granular cells: SGCs; hyalinocyte: HC) (Fig. 1). GCs were the largest cells and contained abundant granules while SGCs were smaller and contained fewer granules than the GCs and were more round in common. HCs were the smallest cells containing few granules, their nucleus occupied most of the room in cells, indicating the largest nuclear-cytoplasmic ratio (N/C). Among the two species of both male and female, their hemocyte sub-population in common shared uniform characters.

### Total hemocyte count (THC)

**Figure 2:**
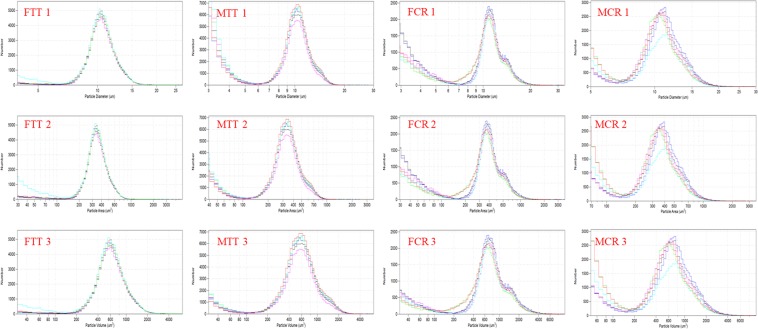
Hemocyte size frequency distribution of *Tachpleus tridentatus* and *Carcinoscorpius rotundicauda* measured by coulter counter (Multisizer 3). 1 indicate hemocyte diameter, expressed in μm, 2 indicate hemocyte area, expressed in μm^2^, and 3 indicate hemocyte volume, expressed in μm^3^; FTT: female *Tachpleus tridentatus*; MTT: male *Tachpleus tridentatus*; FCR: female *Carcinoscorpius rotundicauda*; MCR: male *Carcinoscorpius rotundicauda*.

**Figure 3:**
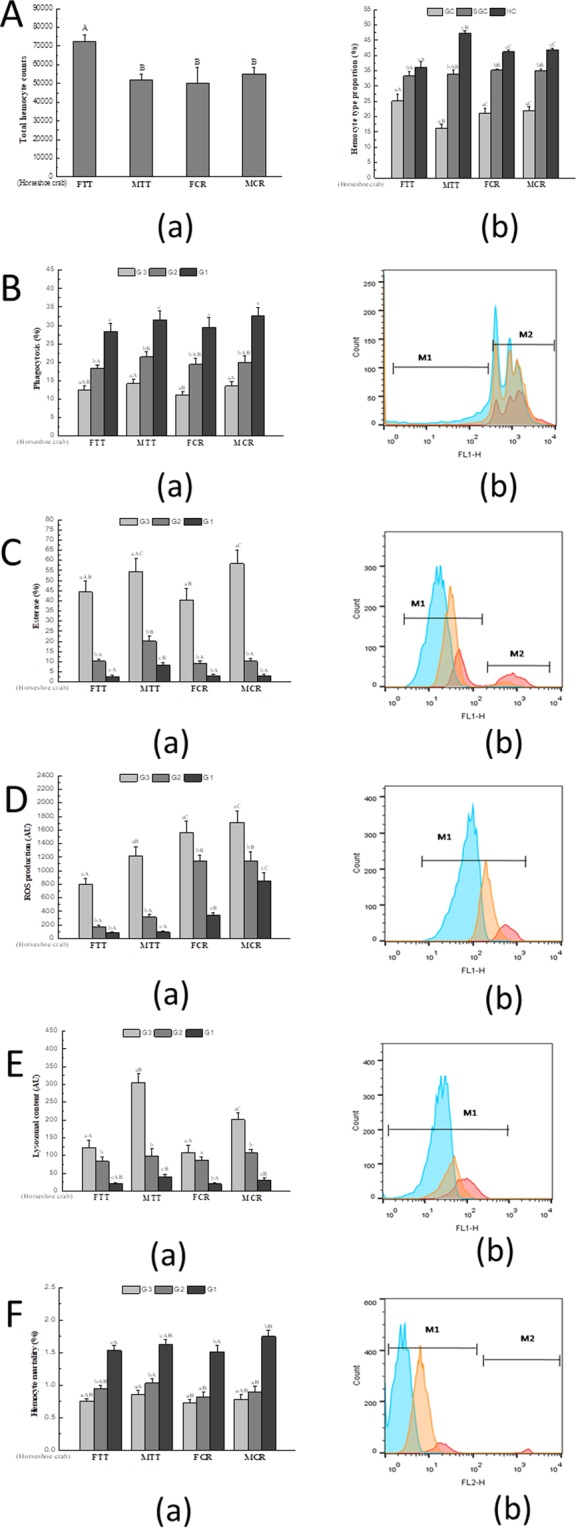
(**A**) (a) Total hemocyte counts (THC), (b) Hemocyte type proportion. (**B**) Phagocytosis (Pha), (**C**) Esterase (Est), (**D**) Reactive oxygen species (ROS), (**E**) Lysosomal content (Lyso), (**F**) Hemocyte mortality (Hm) of *Tachpleus tridentatus* and *Carcinoscorpius rotundicauda* measured by flow cytometry. For (**B**–**F**) (a) is the comparison of the three types of hemocyte, (b) is the frequency histogram showing the relative fluorescence intensities of hemocytes in a log-mode (for **B**,**C**,**F**, M2 indicates higher relative fluorescence intensity compared to M1); blue colour means G1 cell, orange colour means G2 cell, red colour means G3 cell. FTT: female *Tachpleus tridentatus*; MTT: male *Tachpleus tridentatus*; FCR: female *Carcinoscorpius rotundicauda*; MCR: male *Carcinoscorpius rotundicauda*. Small letters indicate significant differences among hemocyte subpopulations (p < 0.05), and capital letters indicate significant differences among four different species (p < 0.05).

Hemocyte size frequency distributions were evaluated by coulter counter, and the size of hemocyte in *T*. *tridentatus* was more centralized than *C*. *rotundicauda* (Fig. 2). For the female *T*. *tridentatus*, the major peak of the hemocyte diameter was observed from 7.5 µm to 17 µm; the major peak of the hemocyte area was at 150 µm^2^–1000 µm^2^; connected with the cell surface area, the hemocyte volume mainly peaked from 150 µm^3^ to 2800 µm^3^. For the male *T*. *tridentatus*, the major peak of the hemocyte diameter was observed from 6 µm to 18 µm; the major peak of the hemocyte area was at 140 µm^2^–1000 µm^2^; connected with the cell surface area, the hemocyte volume mainly peaked from 140 µm^3^ to 2800 µm^3^. For the female *C*. *rotundicauda*, the major peak of the hemocyte diameter was observed from 7 µm to 20 µm; the major peak of the hemocyte area was at 180 µm^2^–1400 µm^2^; connected with the cell surface area, the hemocyte volume mainly peaked from 200 µm^3^ to 4800 µm^3^. For the male *C*. *rotundicauda*, the major peak of the hemocyte diameter was observed from 7.5 µm to 20 µm; the major peak of the hemocyte area was at 150 µm^2^–1400 µm^2^; connected with the cell surface area, the hemocyte volume mainly peaked from 180 µm^3^ to 4600 µm^3^. Among the two species of both male and female, the region and the peak were similar apart from the range of the surface area and volume. The volume of the hemocyte in *C*. *rotundicauda* was larger than those in *T*. *tridentatus*. Total hemocyte counts (THC) of MTT, FCR and MCR (50000–55000 mL^−1^) were significantly (p < 0.05) lower than FTT (ca.72000 mL^−1^, Fig. 3A).

### Hemocyte classification by flow cytometry

**Figure 4:**
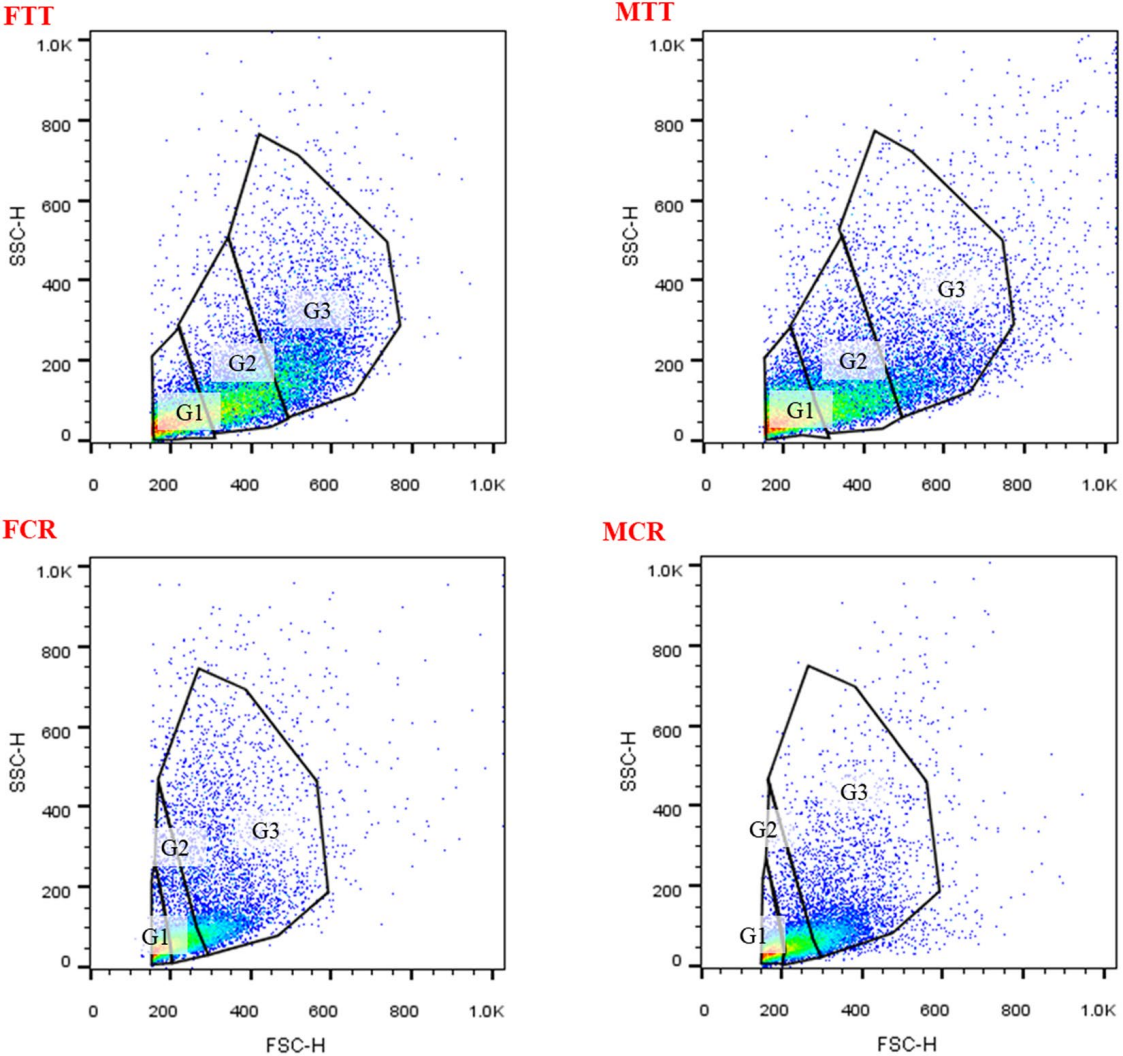
Hemocyte subpopulations of *Tachpleus tridentatus* and *Carcinoscorpius rotundicauda* classified by flow cytometric analysis. Cells were displayed by dot plot, and expressed as cell size (forward scatter, FSC value), versus cell complexity (side scatter, SSC value). Three subpopulations of hemocytes were identified (G1: Gate 1; G2: Gate 2; G3: Gate 3). FTT: female *Tachpleus tridentatus*; MTT: male *Tachpleus tridentatus*; FCR: female *Carcinoscorpius rotundicauda*; MCR: male *Carcinoscorpius rotundicauda*.

At least three subpopulations of hemocytes, in female *T*. *tridentatus* (FTT), male *T*. *tridentatus* (MTT) and female *C*. *rotundicauda* (FCR), male *C*. *rotundicauda* (MCR), were classified, according to the criteria of cell size (FSC) and the cell complexity (SSC, Fig. 4). The gate 1(G1) composes of cells with low complexity and small size, which may mainly include HCs. More complex cells with middle size were classified into gate 2 (G2), which may mainly contain SGCs. Cells in gate 3 (G3) were the biggest and the most complex, probably the GCs, whose proportion of number was the least.

### Immune parameter measurements

For the two horseshoe crabs of both genders, the number of G1 cell was the maximum among three hemocyte types. Then the G2 cell number was more than G3 cell. Among two horseshoe crab species, the proportions of three types of hemocytes displayed some differences. Between FTT and MTT, every group of hemocyte was discrepant, while it showed similarity between FCR and MCR among three types of hemocyte. For all of the species, G1 cells have the highest proportion. All the hemocytes showed phagocytosis capacities, while the proportion of G1 cells was much higher than that of the G3 cells, and G2 cells’ was higher than G3 cells’ but lower than G1 cells’ (Fig. 3B). The esterase activity of males and females of hemocytes did not show prominent difference, while in each species males’ were higher than females’. G3 cells’ were quite higher than G2 cells’, and G2 cells were a bit higher than G1 cells (Fig. 3C). The G3 cells showed higher reactive oxygen species (ROS) and ROS of the G3 cells in MCR and FCR were higher than MTT and FTT (Fig. 3D). The three hemocyte subpopulations had some differences in lysosomal contents, the highest value in the G3 cells, a lower in G2 cells and the lowest in G1 cells (Fig. 3E). Between the two species and between males and females, every three hemocyte subpopulations are almost different. Based on flow cytometry, the total hemocyte mortality was not high, with a mean of less than 4.0%, the mortality of G1 cells measured was significantly higher than G2 and G3 cells (Fig. 3F).

## Discussion

Until now, hemocyte classification in aquatic invertebrate has not reached a uniform standard, but most agree on the classification of granular cells and hyaline cells^35^. Our result showed that the inner structures of hemocytes can be displayed by transmission electron microscope (TEM) and divided into granular cells, semi-granular cells and hyaline cells. According to the number and size of granule, the invertebrate hemocytes could be easily to classify^35^. Three main types of hemocytes were characterized: granular cells, semi-granular cells and hyaline cells in two horseshoe crabs, whose hemocyte types are similar to some other invertebrates, including some crustaceans^35–37^. Besides, the hyaline cells possessed a big nucleus and a few large granules among the Chinese horseshoe crab and mangrove horseshoe crab as some other shellfishes^28,38^. According to the method of optical microscopy, granular cells, semi-granular cells and hyaline cells with different shapes as exhibited were typed for the hemocytes of Chinese horseshoe crab and mangrove horseshoe crab^31^, which are similar with our results. For the two horseshoe crabs, the types and features of hemocytes were similar, and the similar phenomena were also showed in other studies^39^. Besides, hyaline cells were unique contrasting to other cells, which is tally with the study in cultivate horseshoe crab before^30^. Previously, scanning electron microscope) also has been used to view the morphology of the hemocytes in horseshoe crab^31^. Based on the different size and varied numbers of wrinkle on the surface, the hemocytes were classified into three types: granular cells semi-granular cells and hyaline cells^31^.

As an indicator representing the immune functions of hemocyte^40^, total hemocyte counts (THC) were determined in the male and female of the two horseshoe crab species. Among the two species with female and male, the similar results were obtained from the male Chinese horseshoe crab and both male and female of the mangrove horseshoe crab, while the female Chinese horseshoe crab showed some differences. Perhaps different species within the genus as well as different genders can show some differences in such parameter. For example, THCs between the white shrimps *Litopenaeus vannamei* and *Litopenaeus stylirostris* were quite different^41,42^. THCs were also analyzed in two edible crabs, the Jonah crab *Cancer borealis* and the brown crab *Cancer pagurus*, and there were some differences between the two crab species^27^.

In present study, hemocytes also were analyzed by flow cytometry and several indexes were obtained. Usually, the number of hemocyte types varies from species to species. Base on this method, hemocytes in some shellfishes were classified into two^43,44^, three^22,23^ and four^45^ types. FCS and SSC are sometimes not reflecting actual FCS/SCC in crustaceans by FCM^46^, thus the matches between microscopic images and FCM may not be reached exactly. In the present study, hemocytes of the Chinese horseshoe crab and mangrove horseshoe crab, for males and females, were classified into three groups (G1 cells, G2 cells and G3 cells, probably are hyaline cells, semi-granular cells and granular cells). However, the proportion of each type of cell shows differences between two horseshoe crabs.

Phagocytes play a vital role in the innate immune responses in invertebrates^47^. The phagocytosis of hemocytesis poorly defined, it is possible that different types of hemocytes in invertebrates possess different capacities in phagocytosis^48^ or some hemocytes were not phagocytes^22,49^. In the present study, it is obvious that the G1 cells (possible hyaline cells) with less granular were the main phagocytes, whereas G2 and G3 cells had lower phagocytosis, and in the study of Mats^50^, the dominance of hyaline cells in phagocytosis was also observed. While the Chinese mitten crab *Eriocheir sinensis*^43^ showed a different result that G3 cells (possible granulacytes) were the main phagocytes. In our study, G1 cells of the four kinds of horseshoe crab were the main immunocompetent hemocytes, if phagocytosis activity was considered as the major indicator of immunity^50^. Lacking of intracellular prophenoloxidase, hyaline cells could be stimulated by the extracellular prophenoloxidase system, and degranulation has deeply connection with recognition function to foreign objects^51^. The similar result of the phagocytosis parameter was also showed in the mussel *Mytilus edulis*^33^.

The esterase activity of marine invertebrates, like shrimps^52^, mussels^53,54^, and clams^55^ has been extensively reported using flow cytometry. As a kind of hydrolase enzyme, it hydrolyzes some choline esters. This enzyme thus plays an irreplaceable role in the immune defense of marine invertebrates^56,57^, and the mortality of hemocytes increases when esterase activity decreases according to some studies^55,58^. In some studies, the granular cells showed higher esterase activity than hyaline cells and were regarded to the major cell in defense^40,52^, indicating the importance of its granular cells with esterase activity^59^. This was the first time for studying esterase of hemocytes in the Chinese horseshoe crab and mangrove horseshoe crab. For both species, the esterase in G3 cells was higher than that in G1 and G2 cells. That is to suggest that cells with more granules should be more active in esterase activity. Semi-granular cells possess stronger ability for exocytosis, playing an important part in immunity^50,60^.

ROS production, induced by phagocytosis in the process of elimination^61^, is regarded as an important mechanism for microbicidal capacities^62^. ROS is usually used to determine immunity in marine invertebrates, such as bivalves^48,63^ and crustaceans^43,64^. When hemocytes were stimulated (like exposure *in vitro*), respiratory burst occurred and toxic reactive oxygen species were released^65,66^. The most significant substances include superoxide anion (O^2−^), hydrogen peroxide (H_2_O_2_), singlet oxygen (^1^O_2_) and hydroxyl (OH)^67^. In the present study, the ROS production of G3 cells was higher than other hemocytes. The ROS of granulocytes was also higher than that in hyaline cells in the crab *Eriocheir sinensis*^43^. In the hemocytes of *Crassostrea gigas*^23,68^, higher ROS production was detected in granular cells.

Lysosome is an organelle in cells, and lysozymes released from lysosome can non-specifically kill microorganisms by hydrolyzing cell walls^69^. Therefore, lysosome plays key roles in host defenses of microorganisms^70^. In the inactivation of invading microbes, lysozyme participates among the hemolymph constituents^71^. Many studies on hemocytes of marine invertebrates measured lysozyme in hemocyte, such as crabs^43^, oysters^23,72^ and mussels^22,49^. It was found in the green-lipped mussel *P*. *viridis*^40^ that the granular cells contained higher lysosomal content than other groups of hemocytes. Similarly in the present study, lysosomal content in G3 cells was higher than that in G1 cells in the two horseshoe crab species. Cytosolic lysosomes release various enzymes in vesicles, which combines with the production of ROS to destroy the foreign particles^73,74^. Meanwhile, there were some differences in G1 cells among the four kinds of horseshoe crabs, although all of them had lower lysosomal content.

The cellular mortality is usually accompanied by necrotic and apoptotic courses^75^, thus hemocyte mortality is a useful indicator for immune system after being stressed in marine invertebrates^54,76^. There were some studies of hemocytes mortality in mussels^40,63^ and clams^55^. From the present study, hemocyte mortality was lower in G3 cells and higher in G1 cells. Combined with the lysosomal content, higher lysozyme safeguarded lower mortality in G3 cells, and in G1 cells lower lysozyme caused higher mortality, likely that lysosome is relevant to hemocyte mortality tightly owing to the high phagocytosis of G1 cells (hyaline cells) hydrolyzing foreign objects after endocytosis^72^. For example, in the green-lipped mussel *P*. *viridis*^40^, the mortality of hyaline cells was higher than granular cells, indicating that hyaline cells were not positive as granular cells in immune defense. It is possible that different subpopulations are responsible for different functions^48^.

Same as other crustaceans, the immune system of horseshoe crab lacks of immunoglobulin and removes exogenous object and pathogen through phagocytosis activity, esterase activity, ROS production and lysosome, belonging to innate immune^77^. Crustacean hemocytes play important roles in the host immune response including recognition, phagocytosis, melanization, cytotoxicity and cell-cell communication^50,77,78^. Classification of the hemocyte types in decapods crustaceans is based mainly on the presence of cytoplasmic granules in hyaline cells, semi-granular cells, and granular cells^79^. Studies on crustacean have shown that different types of hemocytes play different roles in immunity^50,80^. Each cell type is active in defense reactions, for example, in crayfish, the hyaline cells are chiefly involved in phagocytosis, the semi-granular cells are the cells active in encapsulation, while the granular cells participate in storage and release of the prophenoloxidase and cytotoxicity^50,77,78^.

Apart from those, hemocytes in horseshoe crab need more deeply study. This study has demonstrated that the hemocytes of *C*. *rotundicaud* and *T*. *tridentatus* possess many morphological and functional characteristics as other marine invertebrates, some immune parameters in every types of hemocyte were different to other invertebrates. More studies on immune functions of different subpopulations of hemocytes in horseshoe crab should be further investigated.

## Materials and Methods

### Animals and hemolymph collection

Wild adult horseshoe crabs, *T*. *tridentatus* (male weight: 250.0 ± 20.0 g, female weight: 700.0 ± 10.0 g) and *C*. *rotundicauda* (male weight: 250.0 ± 20.0 g, female weight: 260.0 ± 10.0 g), were collected from the Beibu Gulf, a semi-enclosed sea (17°00′ to 21°45′N and 105°40′ to 110°10′E). During the two-week period of acclimation, 12 *T*. *tridentatus* (male: female = 1:1) and 12 *C*. *rotundicauda* (male: female = 1:1) were fed on the same amount of *Ruditapes philippinarum*, once daily (17:00 h) before experiment, and no horseshoe crabs died. During the acclimation, water temperature was 26 ± 1 °C, pH was 8.1, salinity was 32 ± 1‰, dissolved oxygen (DO) was maintained at 6–8 mg L^−1^ and photoperiod was 12D:12 L with the light period from 06:00 to 18:00.

After the adaptation period, six horseshoe crabs of each gender were sampled to obtain hemolymph for both species. Hemolymph was collected from the joint between bent breastplate and plastron using a 1.0 mL plastic syringe with 22 G needle and all samples obtained were stored in tubes with pre-chilled (187 USP unit ml^−1^) heparin^81^.

### Transmission electron microscopy (TEM)

Five mL of hemolymph were prepared in the solution of 4% paraformaldehyde, 0.3 M sucrose in a 0.1 M cacodylate buffer (pH 7.2) and 2.5% glutaraldehyde for 10 minutes. Then, prepared cells were centrifuged at 400 g at ambient temperature for 10 minutes and the supernatant was discarded. The pellets were resuspended and encased in 3% molten agar. Then the embedded cells were whittled into 1 mm^3^ small blocks and immobilized by fresh fixative at 4 °C overnight. After being washed using cacodylate buffer, cells were fixed with 1% osmium tetroxide (OsO_4_) in 0.1 M cacodylate buffer (pH 7.2) at 4 °C for one hour. Samples were douched in the same cacodylate buffer and distilled water, dehydration in ethanol solutions of six different grades (30%, 50%, 70%, 80%, 95% and 100%) and transferred into acetone. Later before embedding, the samples were infiltrated gradually in resin. Ultrathin sections (60–90 nm) were made by using a Leica Ultracut UCT ultra microtome (Austria). Ultrathin sections were mounted onto coated collodion with 100-mesh copper grids. A section was dyed with 2% uranyl acetate aqueous solution for fifteen minutes before stain with Reynold’s lead citrate for ten minutes. Sections were observed using a FEI/Philips Tecnai 12 BioTWIN transmission electron microscope (Netherlands) operated at 80 kV.

### Total hemocyte counting (THC)

An electronic particle counter/size analyser (Multisizer™ 3 Coulter Counter, Beckman Coulter) was used to evaluate the hemocyte concentration in hemolymph. The size frequency distribution and hemocyte concentration (number of cells per milliliter) were determined. Before sample running, 0.5 mL of hemolymph was added into 9.5 mL of Isoton® II solution to crank out the mixture, and every time 1000 µL of the mixed solution were counted.

### Parameter measurements by flow cytometry

Recently collected hemocytes within unwrought crude hemolymph were analyzed for immune parameters by a BD FACS Calibur flow cytometer equipped with an air-cooled argon laser and offers a laser excitation at a spot of 488 nm. A FSC limitation (>150) was defined to eliminate bacteria and cell debris. Datum was described as cell cytograms pointing the granularity (SSC value), the proportional size (FSC value) and the fluorescence channels identifying with the markers used. For each hemolymph sample, 20000 events were obtained in total, while the flow rate was corrected to maintain the whole events below 300 every second. The fluorescent frequency distribution column diagram of the hemocyte population was subsequently acquired. The type of fluorescence channel relied on the parameter monitored: hemocyte mortality was evaluated by FL2 (greenish orange emission for 585 nm), while reactive oxygen species (ROS), enzymes and phagocytosis were measured using FL1 (green emission for 530 nm). Data were analyzed by FlowJo^®^ 10.0 software.

Hemocyte mortality was detected by propidium iodide (PI), which is a type of fluorescent dye that only enters and stains dead hemocytes. 10 μL of solution of PI (Siama Aldrich) at a concentration of l mg·mL^−1^ was added into 400 μL hemolymph, and the mixture was incubated for 30 minutes in the dark at 4 °C before analyzed by flow cytometry. Hemocyte mortality was analyzed as the percentage of hemocytes showing PI fluorescence relative to the whole hemocyte counts.

Phagocytosis was detected using fluorescent microspheres as a phagocytic goal by an in italic assay, and estimated as the percentage of cells having internalized at least three fluorescent beads^40,82^. 400 μL of hemolymph was incubated for 1 hour at ambient temperature in the dark with 10 μL of the Fluorospheres^®^ carboxylate-modified microspheres with 1/10 dilution (yellow-green fluorescent, 1 μm diameter, Invitrogen). Finally, the fluorescent beads concentration was 10^8^ mL^−1^, and the beads/hemocytes ratio was 100/1.

Activity of non-specific esterase was detected using non-specific lip soluble ground substance fluorescein diacetate (FDA, Sigma). FDA stock solutions (0.04 mM) were prepared in dimethyl sulphoxide (DMSO) and reserved at −20 °C. Working solutions of FDA (400 μM) were drew up by 1/10 diluting the stock with strained and sterile seawater. 400 microliters of hemolymph with 2 μL FDA solution was incubated in the dark for 15 minutes at ambient temperature. The percentage of cells expressing enzymatic activity was defined based on the percentage of fluorescent cells among all cells.

The cellular reactive oxygen species (ROS) were measured by 2′7′-dichlorofluorescein diacetate (DCFH-DA; Sigma). DCFH-DA, a nonfluorescent fluorescein analogue, can disseminate into hemocytes, and then is hydrolysed into 2′,7′-dichlorofluorescein (DCFH). Enzymatic activity was defined based on fluorescent cells among the whole cells. A 10 mM DCFH-DA stock was added to DMSO and is generally diluted to 10% in strained sterile seawater as a working fluid. Each analysis required mixture with four hundred microliters of hemolymph and 4 μL of DCFH-DA, and the mixture was then incubated in the dark for 15 minutes at ambient temperature.

Lysosomal content was measured by a merchant LysoTracker^®^ Yellow HCK-123 (1 mM in DMSO, Invitrogen). 400 μL hemolymph mixed with 1 μL of LysoTracker were incubated took place at ambient temperature for two hours in the dark and the reaction was ceased on ice. LysoTracker fluorescence in the hemocytes was described in arbitrary units (A.U.).

### Statistics analysis

Prior to the analysis, data were checked for homogeneity of variance with Levene’s test and normality with the Shapiroe-Wilk’s test using statistical software SPSS 18.0. Percentage data were arcsine transformed. One-way analysis of variance (ANOVA) and Tukey’s test were used to compare the differences of immune parameters among granular cell, semi-granular cell and hyaline cell identified by flow cytometry. For all analysis, the results are expressed as the means ± SD of the data and significant differences were known as p < 0.05.

## References

[1] Hu MH, Wang YJ, Tsang ST, Cheung SG, Shin PKS (2011). Effect of starvation on the energy budget of two Asian horseshoe crab species: *Tachypleus tridentatus* and *Carcinoscorpius rotundicaud*a (Chelicerata: Xiphosura). Marine Biology.

[2] Chen CP (2015). Co-occurrence of juvenile horseshoe crabs *Tachypleus tridentatus* and *Carcinoscorpius rotundicauda* in an estuarine bay, southwestern China. Aquatic Biology.

[3] Kwan BKY, Hsieh H-L, Cheung SG, Shin PKS (2016). Present population and habitat status of potentially threatened Asian horseshoe crabs *Tachypleus tridentatus* and *Carcinoscorpius rotundicauda* in Hong Kong: a proposal for marine protected areas. Biodiversity and Conservation.

[4] Kwan BKY, Chan AKY, Cheung SG, Shin PKS (2017). Marine microalgae as dietary supplements in the culture of juvenile Chinese horseshoe crabs, *Tachypleus tridentatus* (Xiphosura). Aquaculture Research.

[5] Beisel HG, Kawabata S, Iwanaga S, Huber R, Bode W (1999). Tachylectin-2: crystal structure of a specific GlcNAc/GalNAc-binding lectin involved in the innate immunity host defense of the Japanese horseshoe crab *Tachypleus tridentatus*. Embo Journal.

[6] Srijaya TC (2014). Colour preference and light sensitivity in trilobite larvae of mangrove horseshoe crab, *Carcinoscopius rotundicauda* (Latreille, 1802). Indian Journal of Experimental Biology.

[7] Attaya Kungsuwan YN (1987). Tetrodotoxin in the Horseshoe Crab *Carcinoscorpius rotundicauda* Inhabiting Thailand. Nippon Suisan Gakkaishi.

[8] Bandyopadhyay R, Basu MK (1988). Phospholipids from the hepatopancreas of Indian horseshoe crab *Carcinoscropius rotundicauda*. Biochimie.

[9] Tanu MB, Noguchi T (1999). Tetrodotoxin as a toxic principle in the horseshoe crab *Carcinoscorpius rotundicauda* collected from Bangladesh. Shokuhin Eiseigaku Zasshi.

[10] Dao HV, Takata Y, Sato S, Fukuyo Y, Kodama M (2009). Frequent occurrence of the tetrodotoxin-bearing horseshoe crab *Carcinoscorpius rotundicauda* in Vietnam. Fisheries Science.

[11] Cartwright Taylor L, Bing YV, Chi HC, Tee LS (2011). Distribution and abundance of horseshoe crabs *Tachypleus gigas* and *Carcinoscorpius rotundicauda* around the main island of Singapore. Aquatic Biology.

[12] Adibah AB, Ling LP, Tan SG, Faridah QZ, Christianus A (2012). Development of single-locus DNA microsatellite markers using 5′anchored ISSR-PCR method for the mangrove horseshoe crab, *Carcinoscorpius rotundicauda* (Latreille, 1802) in Peninsular Malaysia. Molecular Biology Reports.

[13] Widener JW, Barlow RB (1999). Decline of a horseshoe crab population on Cape Cod. Biological Bulletin.

[14] Lee CN, Morton B (2016). Changes in the distributions of juvenile horseshoe crabs (*Arthropoda: Chelicerata*) (2002–2014) related to environmental perturbations at Pak Nai and Ha Pak Nai, Deep Bay, Hong Kong SAR, China. Marine Pollution Bulletin.

[15] Hu MH (2009). Summer distribution and abundance of juvenile Chinese horseshoe crabs *Tachypleus tridentatus* along an intertidal zone in southern China. Aquatic Biology.

[16] Botton ML, Shuster CN, Sekiguchi K, Sugita H (1996). Amplexus and mating behavior in the Japanese horseshoe crab, *Tachypleus tridentatus*. Zoological Science.

[17] Brown KL, Hancock REW (2006). Cationic host defense (antimicrobial) peptides. Current Opinion in Immunology.

[18] Hancock REW, Brown KL, Mookherjee N (2006). Host defence peptides from invertebrates - emerging antimicrobial strategies. Immunobiology.

[19] Silva NC, Sarmento B, Pintado M (2013). The importance of antimicrobial peptides and their potential for therapeutic use in ophthalmology. International Journal of Antimicrobial Agents.

[20] Muta T, Iwanaga S (1996). The role of hemolymph coagulation in innate immunity. Current Opinion in Immunology.

[21] Sun R (2013). Hemocytic immune responses triggered by CpG ODNs in shrimp *Litopenaeus vannamei*. Fish & Shellfish Immunology.

[22] Evariste L (2016). Functional features of hemocyte subpopulations of the invasive mollusk species *Dreissena polymorpha*. Fish & Shellfish Immunology.

[23] Wang WL (2017). The granulocytes are the main immunocompetent hemocytes in *Crassostrea gigas*. Developmental and Comparative Immunology.

[24] Novitsky TJ (1984). Discovery to commercialization: the blood of the horseshoe crab. Oceanus.

[25] Andreu, D. & Rivas, L. Animal antimicrobial peptides: An overview. *Biopolymers* **47**, 415–433, https://doi.org/10.1002/(sici)1097-0282(1998)47:6<415::aid-bip2>3.0.co;2-d (1998). 10.1002/(SICI)1097-0282(1998)47:6<415::AID-BIP2>3.0.CO;2-D 10333735

[26] Harnedy PA, FitzGerald RJ (2012). Bioactive peptides from marine processing waste and shellfish: A review. Journal of Functional Foods.

[27] Parrinello D, Sanfratello MA, Celi M, Vazzana M (2015). Hemocyte types and some plasmatic properties of two edible crabs *Cancer borealis* and *Cancer pagurus*. *Isj-Invertebrate Survival*. Journal.

[28] Li SG (2015). Morphology and classification of hemocytes in *Pinctada fucata* and their responses to ocean acidification and warming. Fish & Shellfish Immunology.

[29] Preziosi BM, Bowden TJ (2016). Morphological characterization via light and electron microscopy of Atlantic jackknife clam (*Ensis directus*) hemocytes. Micron.

[30] Hsieh MC (2011). Comparison of Fixed and Stained Hemocytes from *Cultivate Horseshoe Crab* in Taiwan. Journel of Taiwan Fish. Research..

[31] Wu, F. L. *et al*. Classification and characterization of hemocytes between *Tachpleus tridentatus* and *Carcinoscorpius rotundicauda*. *Acta Hydrobiologica Sinica* **39**, 1169–1176 (2011). (In Chinese with English abstract).

[32] Ren XY, Gao BQ, Liu XX, Li J, Liu P (2017). Comparison of immune responses and antioxidant status of different generations of growth-selected *Portunus trituberculatus* families. Aquaculture Research.

[33] Caza F (2015). Comparative analysis of hemocyte properties from *Mytilus edulis desolationis* and *Aulacomya ater* in the Kerguelen Islands. Marine Environmental Research.

[34] Pezzati E (2015). Susceptibility of Vibrio aestuarianus 01/032 to the antibacterial activity of *Mytilus* haemolymph: identification of a serum opsonin involved in mannose-sensitive interactions. Environmental Microbiology.

[35] Vázquez Lorena, Pérez Armando, Millán Diana, Agundis Concepción, Martin Gary, Cooper Edwin L., Lascurain Ricardo, Zenteno Edgar (1997). Morphology of hemocytes from the freshwater prawnMacrobrachium rosenbergii. Journal of Morphology.

[36] Giulianini PG, Bierti M, Lorenzon S, Battistella S, Ferrero EA (2007). Ultrastructural and functional characterization of circulating hemocytes from the freshwater crayfish *Astacus leptodactylus*: Cell types and their role after *in vivo* artificial non-self challenge. Micron.

[37] Martin GG, Castro C, Moy N, Rubin N (2003). N-acetyl-D-glucosamine in crustacean hemocytes; possible functions and usefulness in hemocyte classification. Invertebrate Biology.

[38] Lv SJ (2014). Classification and phagocytosis of circulating haemocytes in Chinese mitten crab (*Eriocheir sinensis*) and the effect of extrinsic stimulation on circulating haemocytes *in vivo*. Fish & Shellfish Immunology.

[39] Kondo M, Yasumoto S, Takahashi Y (2016). Classification of Hemocytes in Four Species of Land Hermit Crabs (*Coenobita*) and Coconut Crab (*Birgus latro*): A New Classification Group of Polyhemocytic Crustaceans Found in Coconut Crab. Journal of National Fisheries University.

[40] Wang YJ, Hu MH, Chiang MWL, Shin PKS, Cheung SG (2012). Characterization of subpopulations and immune-related parameters of hemocytes in the green-lipped mussel *Perna viridis*. Fish & Shellfish Immunology.

[41] Goarant C, Boglio E (2000). Changes in hemocyte counts in *Litopenaeus stylirostris* subjected to sublethal infection and to vaccination. Journal of the World Aquaculture Society.

[42] Li B (2014). Effect of temperature decrease on hemocyte apoptosis of the white shrimp *Litopenaeus vannamei*. Aquaculture International.

[43] Jia ZH (2017). Functional characterization of hemocytes from Chinese mitten crab *Eriocheir sinensis* by flow cytometry. Fish & Shellfish Immunology.

[44] Koiwai K (2017). Two hemocyte sub-populations of kuruma shrimp *Marsupenaeus japonicus*. Molecular Immunology.

[45] Garcia-Garcia E, Prado-Alvarez M, Novoa B, Figueras A, Rosales C (2008). Immune responses of mussel hemocyte subpopulations are differentially regulated by enzymes of the PI3-K, PKC, and ERK kinase families. Developmental and Comparative Immunology.

[46] Li F, Chang X, Xu L, Yang F (2018). Different roles of crayfish hemocytes in the uptake of foreign particles. Fish & Shellfish Immunology.

[47] Jiang S (2016). Functional characterisation of phagocytes in the Pacific oyster *Crassostrea gigas*. Peerj.

[48] Lau Y, Sussman L, Pales Espinosa E, Katalay S, Allam B (2017). Characterization of hemocytes from different body fluids of the eastern oyster *Crassostrea virginica*. Fish & Shellfish Immunology.

[49] Yang H (2015). Morphology and Immune-related activities of hemocytes of the mussel *Mytilus coruscus* (Gould, 1861) from East Sea of Korea. Ocean Science Journal.

[50] Johansson MW, Keyser P, Sritunyalucksana K, Soderhall K (2000). Crustacean haemocytes and haematopoiesis. Aquaculture.

[51] Söderhäll K, Smith VJ, Johansson MW (1986). Exocytosis and uptake of bacteria by isolated haemocyte populations of two crustaceans: evidence for cellular co-operation in the defence reactions of arthropods. Cell & Tissue Research.

[52] Xian JA, Zhang XX, Guo H, Wang DM, Wang AL (2016). Cellular responses of the tiger shrimp *Penaeus monodon* haemocytes after lipopolysaccharide injection. Fish & Shellfish Immunology.

[53] Huang XZ (2016). Hemocyte responses of the thick shell mussel *Mytilus coruscus* exposed to nano-TiO_2_ and seawater acidification. Aquatic Toxicology.

[54] Wu FL (2016). Combined effects of seawater acidification and high temperature on hemocyte parameters in the thick shell mussel *Mytilus coruscus*. Fish & Shellfish Immunology.

[55] Gajbhiye DS, Khandeparker L (2017). Immune response of the short neck clam *Paphia malabarica* to salinity stress using flow cytometry. Marine Environmental Research.

[56] Lehtonen KK (2006). The BEEP project in the Baltic Sea: Overview of results and outline for a regional biological effects monitoring strategy. Marine Pollution Bulletin.

[57] Xian JA (2012). *In vitro* toxicity of nitrite on haemocytes of the tiger shrimp, *Penaeus monodon*, using flow cytometric analysis. Comparative Biochemistry and Physiology C-Toxicology & Pharmacology.

[58] Gagnaire B, Frouin H, Moreau K, Thomas-Guyon H, Renault T (2006). Effects of temperature and salinity on haemocyte activities of the Pacific oyster, *Crassostrea gigas* (Thunberg). Fish & Shellfish Immunology.

[59] Chang SJ, Tseng S, Chou H (2013). Cytochemical and Immunological Studies of Hemocytes in Portuguese Oyster, *Crassostrea angulata*. Journel of Taiwan Fish. Research.

[60] Martin GG, Graves BL (1985). Fine structure and classification of shrimp hemocytes. Journal of Morphology.

[61] Newton K, Dixit VM (2012). Signaling in innate immunity and inflammation. Cold Spring Harbor perspectives in biology.

[62] Terahara K, Takahashi KG (2008). Mechanisms and immunological roles of apoptosis in molluscs. Current Pharmaceutical Design.

[63] Ben Cheikh Y (2016). First evidence for a Vibrio strain pathogenic to *Mytilus edulis* altering hemocyte immune capacities. Developmental and Comparative Immunology.

[64] Xu HS (2015). Effect of lipopolysaccharide on the hemocyte apoptosis of *Eriocheir sinensis*. Journal of Zhejiang University-Science B.

[65] Bell KL, Smith VJ (1993). *In vitro* superoxide production by hyaline cells of the shore crab Carcinus maenas (L.). Developmental and Comparative Immunology.

[66] Munoz M (2000). Measurement of reactive oxygen intermediate production in haemocytes of the penaeid shrimp, Penaeus vannamei. Aquaculture.

[67] Feng SY, Feng JS, Burke CN, Khairallah LH (1971). Light and electron microscopy of the leucocytes of Crassostrea virginica (Mollusca: Pelecypoda). Zeitschrift Für Zellforschung Und Mikroskopische Anatomie.

[68] Jiang S (2016). The cytochemical and ultrastructural characteristics of phagocytes in the Pacific oyster *Crassostrea gigas*. Fish & Shellfish Immunology.

[69] Monari M (2007). Effects of high temperatures on functional responses of haemocytes in the clam *Chamelea gallina*. Fish & Shellfish Immunology.

[70] Ciechanover A (2005). Proteolysis: from the lysosome to ubiquitin and the proteasome. Nature Reviews Molecular Cell Biology.

[71] Mohandas A (1985). An electron microscope study of endocytosis mechanisms and subsequent events in mercenaria mercenaria granulocytes. Parasitic and Related Diseases.

[72] Cho SM, Jeong WG (2005). Spawning impact on lysosomal stability of the Pacific Oyster, *Crassostrea gigas*. Aquaculture.

[73] Carballal MJ, Lopez MC, Azevedo C, Villalba A (1997). Hemolymph cell types of the mussel Mytilus galloprovincialis. Diseases of Aquatic Organisms.

[74] Donaghy L, Lambert C, Choi K, Soudant P (2009). Hemocytes of the carpet shell clam (Ruditapes decussatus) and the Manila clam (Ruditapes philippinarum): Current knowledge and future prospects. Aquaculture.

[75] Goedken M, De Guise S (2004). Flow cytometry as a tool to quantify oyster defence mechanisms. Fish & Shellfish Immunology.

[76] Bouilly K (2006). Effects of cadmium on aneuploidy and hemocyte parameters in the Pacific oyster, *Crassostrea gigas*. Aquatic Toxicology.

[77] Soderhall, K. & Thornqvist, P. O. In *Fish Vaccinology* Vol. 90 *Developments in Biologicals* (eds Gudding, R., Lillehaug, A., Midtlyng, P. & Brown, F.) 45–51 (1997).

[78] Söderhäll K, Cerenius L (1998). Role of the prophenoloxidase-activating system in invertebrate immunity. Current Opinion in Immunology.

[79] Bauchau, A. G. *Crustaceans*. *In: Ratcliffe*. 385–420 (1980).

[80] Hose JE (1990). A Decapod Hemocyte Classification Scheme Integrating Morphology, Cytochemistry, and Function. Biological Bulletin.

[81] Kwan BKY, Chan AKY, Cheung SG, Shin PKS (2014). Hemolymph quality as indicator of health status in juvenile Chinese horseshoe crab Tachypleus tridentatus (Xiphosura) under laboratory culture. Journal of Experimental Marine Biology and Ecology.

[82] Gagnaire B, Thomas-Guyon H, Burgeot T, Renault T (2006). Pollutant effects on Pacific oyster, Crassostrea gigas (Thunberg), hemocytes: Screening of 23 molecules using flow cytometry. Cell Biology and Toxicology.

